# Erratum for Earnshaw et al., “Real-World Financial and Clinical Impact of Diagnostic-Driven and Empirical-Treatment Strategies in High-Risk Immunocompromised Patients with Suspected Aspergillus Infection in the United Kingdom”

**DOI:** 10.1128/spectrum.03031-22

**Published:** 2022-08-18

**Authors:** Stephanie R. Earnshaw, Cheryl McDade, Andrew Bryan, Monica Ines, Christianne Micallef, Anita Sung, David A. Enoch

**Affiliations:** a RTI Health Solutions, Research Triangle Park, North Carolina, USA; b Pfizer Biopharmaceuticals Group, Pfizer Ltd., Surrey, United Kingdom; c Hospital & Vaccines Business Unit, Pfizer, Inc., Porto-Salvo, Portugal; d Cambridge University Hospitals NHS Foundation Trust, Cambridge, United Kingdom; e Pfizer, Inc., New York, New York, USA

## ERRATUM

Volume 10, no. 3, e00425-22, 2022, https://doi.org/10.1128/spectrum.00425-22. Page 3, Table 1, column 4: “−£441” should read “−£681,” and “£680” should read “£227.”

